# Trajectories of Physical Activity Among Adolescents in the Transition From Primary to Secondary School

**DOI:** 10.3389/fspor.2020.00085

**Published:** 2020-08-04

**Authors:** Hilde Kristin Mikalsen, Marte Bentzen, Reidar Säfvenbom, Pål Aril Lagestad

**Affiliations:** ^1^Department of Physical Education and Sport Science, Nord University, Levanger, Norway; ^2^Department of Teacher Educations and Outdoor Studies, The Norwegian School of Sport Sciences, Oslo, Norway

**Keywords:** accelerometer, eagerness for physical activity (EPA), perceived athletic competence (PAC), parental support (PPS), longitudinal

## Abstract

Research on physical activity (PA) behavior reveals an overall decrease worldwide from early childhood and throughout adulthood. The ability to illuminate which factors promote activity for whom and in which phase of life, therefore, becomes a key concept in extending our understanding of individuals' physical activity trajectories. Accordingly, this study investigates latent trajectories of objectively measured PA in adolescents (*n* = 306) over 3 years from ages 13 to 15. Further, it was tested whether eagerness for physical activity, perceived athletic competence, and parental support were associated with the different trajectories of PA. Latent class growth analysis revealed two PA trajectories (trajectory 1: “decrease from very high” and trajectory 2: “steeper decrease from high”). Trajectory 1 had a higher PA level at baseline and less PA decline than trajectory 2. Trajectory 1, “decrease from very high,” was associated with higher levels of eagerness for physical activity and perception of competence at all three time points. Furthermore, the effect size of differences between trajectory 1, “decrease from very high,” and trajectory 2, “steeper decrease from high,” increased from baseline (age 13, seventh grade) to posttest (age 15, ninth grade). This finding indicates a stronger experience of PA as enjoyable, personally relevant, and self-confirming behavior (i.e., “I regard myself as a person who exercises”) within the most active adolescents and even stronger as they get older. Consistent with previous research, the PA level declined from seventh to ninth grade. Being more eager for PA and perceiving oneself more as athletically competent is related to higher levels of PA. This highlights the importance of optimizing environmental factors that increase adolescents' experience of eagerness for physical activity and physical athletic competence.

## Introduction

Although lack of physical activity (PA) and extensive and uninterrupted sitting are known risk factors for major, non-communicable diseases (Trost et al., 2014; WHO, 2014; Reis et al., 2016), a sufficient level of PA has shown to have the potential to reduce health risks, empower natural development and learning, and increase overall quality of life (Pescatello et al., 2014; Helsedirektoratet, 2016; Poitras et al., 2016). The importance of PA in young people's lives has been highlighted for many years both internationally (WHO, 2009, 2018) and nationally by governments in several countries (Helsedirektoratet, 2016), and PA interventions have been carried out according to governmental recommendations. Despite these efforts, research on PA behavior reveals an overall decrease worldwide from early childhood and throughout adulthood (Dumith et al., 2011; Reilly, 2016; HuNT, 2019). Consequently, too many adolescents, all over the world, are still not fulfilling national PA recommendations (Recours et al., 2011; Ruotsalainen et al., 2015; WHO, 2018; Steene-Johannesen et al., 2019).

Tracking studies have shown that early life PA habits seem to be a significant predictor of PA behavior later in life even if people's PA levels fluctuate through different periods of life and that the stability of PA seems to be lower in transitional phases, such as from childhood to adolescence (Baxter-Jones et al., 2005; Telama et al., 2014; Rauner et al., 2015; Reilly, 2016; Rovio et al., 2018; Štefan et al., 2018; Hayes et al., 2019). By using latent class growth analysis (LCGA) in a longitudinal exploration (31 years; *N* = 3,596), Rovio et al. additionally revealed developmental differences within a sample consisting of individuals who were 9 years old at baseline by identifying five different PA trajectories. They identified group 1 (6.6%), who remained persistently active; group 2 (13.9%) with a decreasing PA level; group 3 (13.5%) with an increasing PA level; group 4 (51.4%), who were persistently less active (reference group); and group 5 (14.6%), who were persistently inactive.

According to previous studies aiming to identify why some adolescents are physically active and others not, it can be claimed that some variables (i.e., age, sex, self-efficacy, previous PA, access to facilities) can partially explain the phenomenon, but no variables can explain PA levels among adolescents completely (Bauman et al., 2012; Brug et al., 2017; Martins et al., 2017; Rowlands, 2018). PA behavior may, thus, be regarded as an outcome of complex, dynamic, and interactive individual–context relations. The ability to illuminate which factors promote activity for whom and in which phase of life becomes, therefore, a key concept in extending our understanding of individuals' physical activity trajectories (Atkin et al., 2016). Based on what is mentioned above, the aim of this study is 2-fold. First, we identify potential PA trajectories in the sample of adolescents from seventh through ninth grade as a behavioral (biological) system component. Second, in order to extend our understanding of the PA trajectories, we aimed to examine them in relation to three bio-psycho-social concepts: the concept of eagerness for physical activity (EPA), perceived athletic competence (PAC), and perception of parental support (PPS). In addition, sex is considered as a covariate. The intended study variables have, in a previous baseline study, been proven to impact the PA level among adolescents (Mikalsen et al., 2019). According to Rovio et al. (2018), who argue for the importance of pointing out how changes in determinants through the life course affect PA trajectories, we consider a longitudinal follow-up examination of these variables to be an important contribution to the scientific literature.

The concept of *eagerness* reflects a positive mental state, characterized by enjoyment, passion, and even a deeply felt longing or desire for something that does one good (Säfvenbom et al., 2016). Applying this concept in terms of physical activity, eagerness for physical activity implies positive physical activity behavior in contrast to physical activity behavior aiming to prevent negative health outcomes. King and Gaerlan (2014) and Higgins et al. (2003), in previous research, consider enjoyment and desire as positive-activating emotions, and Pekrun et al. (2011) find these positive mental states to be related to both interest in phenomena, effortful behaviors, use of deep strategies, self-regulation, and learning. According to Säfvenbom et al. (2016), the concept of eagerness and, thus, EPA is rooted theoretically in Dewey's theories of experience and considered to be the outcome of continuous and reciprocal individual–world relations (Dewey, 2008; Agans et al., 2013; Säfvenbom et al., 2016). The interactive experiences that grow out of these relations constitute the individual's reference and assessment base when encountering new bodily experiences. Every new action is, thus, to be understood as a product of an interrelated process of personal experiences, self-organization, and valid intentions and hopes for further actions. With this point of departure, individuals can be regarded as products yet also active producers of their ontogeny (Brandtstädter, 2006) or as “co-developers of their developmental pathways, adaptively responding to different biological, social, cultural, and physical environmental contexts that they influence and are also influenced by” (Wood et al., 2018, p. 124). In a previous study (Mikalsen et al., 2019), EPA is identified to be a significant correlate of PA among young adolescents.

*Perception of being competent* is described as important mental capital in the context of development and learning (Deci and Ryan, 2015; Horn, 2015). PAC is referred to by Weiss and Phillips (2015) as a person's “beliefs, judgments, and feelings about one's physical abilities and competencies in general, or in a particular domain.” In addition to the perspective of PAC as a primarily psychological concept (Ommundsen, 2008), others point to the fact that perception of competence is a construction made by an interactive individual–cultural understanding of what to be competent in and which level of competence is socially acceptable in the local PA context (Larsson and Quennerstedt, 2012; Larsson, 2016). Through extended perspective-taking skills as children grow into adolescence, sensitivity to mechanisms, such as social comparison, increase (Harter, 2012). Individuals accordingly advance their ability to make assessments of one's competence and make their positive self-appraisal more vulnerable and their self-protecting needs more prominent (Harter, 2012). PAC is identified in several studies as being a significant correlate of PA, especially among children and adolescents (Timo et al., 2016; Mikalsen et al., 2019). In the current study, PAC is, therefore, explored as an age-dependent psycho-social construction that influences future actions based on a basic human urge to protect and enhance one's self-perception.

From the perspective of all young individuals being both active and acted upon within their environmental contexts (Lerner and Overton, 2008), parents are presumed to impact significantly on their offspring's developmental processes, i.e., establishing physical activity behavior (McDavid et al., 2011; Gerard and Booth, 2015; Weiss and Phillips, 2015; Koning et al., 2016). Furthermore, *parental influence*, consisting of intangible (motivation and information) and tangible (instrumental and conditional) influential categories (Beets et al., 2010), as perceived by the adolescent, is revealed to be an important aspect of parental impact on adolescents' PA behavior (Glozah and Pevalin, 2015; Mikalsen et al., 2019). Applying a developmental perspective, Fredricks and Eccles (2004) suggest that parental influence differs according to developmental stage. On the other hand, previous research indicates that parents' impact on their offspring's PA behavior seems to extend beyond adolescence (Norton et al., 2003; Lam and McHale, 2015), only affected by a temporary period of lower parental influence on PA behavior during early adolescence (age 10–12 years). A possible explanation for this fluctuation, as discussed by Pugliese and Tinsley (2007), is that parents may not represent as salient models for early adolescents concerning PA behaviors when compared with models offered by peers. Other studies (Pugliese and Tinsley, 2007; Beets et al., 2010) reveal that parental influence is mutually related to their offspring's involvement in PA, indicating a possible decrease following the decreasing PA level during childhood and adolescence (Steene-Johannesen et al., 2019). On the basis of the various findings of parental impact on their offspring's PA behavior, PPS is explored as a distal outcome variable in relation to the participants' PA behavior from years 13 to 15.

Previous research finds *sex* to be a rather consistent predictor of physical activity behavior (Bauman et al., 2012). However, more recent studies show some inconsistency according to sex in combination with age. A study by Eberline et al. (2018) finds no sex differences in PA level among American fifth-graders. Nor did Mikalsen et al. (2019) find such differences among Norwegian seventh-graders. On the other hand, the Norwegian mapping studies of PA level (Steene-Johannesen et al., 2019) do reveal higher levels of moderate PA in 6- and 9-year-old boys than girls. Sex differences at 15 years old were only revealed in vigorous PA (boys > girls). American studies of physical activity behavior (Kohl and Cook, 2013) find sex differences in PA level from the age of 6, differences that increase with age. In our study, sex is of no primary interest, but due to uncertainties regarding the impact of sex on 13- to 15-year-old adolescents' PA behavior, sex is applied as a sociodemographic covariate in our analysis.

## Aims of Study

Improving our knowledge about which factors promote activity for whom and in which phase of life, Bauman et al. (2012) and Reilly (2016) call for longitudinal studies. This study's design can be assessed as an appropriate reply to their call, addressing longitudinal explorations of changes in moderate-to-vigorous PA (MVPA) with objective measures made at multiple time points, crossing childhood and adolescence and, further, exploring how eagerness for physical activity, as a less studied variable in relation to physical activity, is associated with different trajectories of physical activity.

With this point of departure, the specific aims of the present study are 2-fold: (1) to explore valid PA trajectories within the total sample of adolescents from ages 13 to 15. This time period of interest includes the transition from primary to secondary school, which is regarded to be a critical phase with regard to lifestyle habits (Eccles and Roeser, 2011; Reilly, 2016). (2) We aim to examine how EPA, PAC, and PPS, referred to as distal outcome variables (Asparouhov and Muthèn, 2015), are associated with different PA trajectories.

## Materials and Methods

### Participants and Procedures

The data material in this longitudinal study comprises accelerometer measures and questionnaire surveys from mid-Norwegian adolescents, who transitioned from primary to secondary school during the data-collection period. The first time point for survey and accelerometer measurement (T1) was in the seventh grade (12–13 years old) at primary school (April 2017), the second data collection (T2) was in the eighth grade (13–14 years old) at secondary school (April 2018), and the last data collection (T3) was in their ninth grade (14–15 years old) (April 2019). At the first measurement time point (April 2017), the sample of 320 adolescents (77% of the cohort) consisted of 161 girls and 159 boys. They belonged to all of the 18 primary schools in two medium-sized municipalities (ca. 15,000–22,000 inhabitants). The two municipalities were chosen using a stratified selection. At the second and third measurement time points, the participants belonged to all of the four secondary schools located in the same two municipalities. We assess our sample to be representative of other samples located in other medium-sized municipalities, including adolescents from both cities and more rural areas. However, by not using a randomized selection of municipalities, we cannot make any conclusions about the representativity of our results.

In cooperation with the municipalities' school governments and the school teachers, invitations to participate in the study were distributed. Both the parents and adolescents gave their written informed consent to participate. All three surveys were conducted in the participants' classrooms during school time. The participants were asked to answer the same questionnaire at all measurement time points. The surveys and the accelerometer measurements were conducted during the same period. The adolescents were informed they would be included in a drawing for a reward after participating in all three data-collection periods. The study has been approved by the Norwegian Center for Research Data (NSD), and it was conducted in accordance with ethical standards for research set by NSD.

### Measures

In accordance with both national (Helsedirektoratet, 2016) and international (WHO, 2018) recommendations for physical activity levels for children and young persons, physical activity level in the present study is presented as MVPA and measured using an accelerometer (Actigraph GT1M). Operationalizing physical activity levels into MVPA makes data comparable to previous studies (Guinhouya et al., 2013; Hayes et al., 2019; Steene-Johannesen et al., 2019). The adolescents were instructed to wear the accelerometer on their right hip for 7 days consecutively except during water activities or while sleeping. According to the test protocol, a daily wear-time of 8 h for a minimum of 2 days was set as a criterion for a valid measurement (Steene-Johannesen et al., 2019). The activity level was registered as counts per minute (cpm), and average cpm for valid days (≥2) was applied. Cutoff for MVPA was also set in line with a Norwegian population study (Steene-Johannesen et al., 2019) with intervals of 2,000 counts or more. Periods with zero registrations for more than 20 min and the period between 12:00 and 6:00 am were not included.

The different variables in the questionnaire are constituted by previously validated scales. The questions in the scales have closed response alternatives designed with 4 or 7 Likert scale alternatives (Ringdal, 2013) with neutral middles in the seven-option scales.

EPA was measured employing the “Eagerness for Physical Activity Scale” (EPAS) (Säfvenbom et al., 2016). EPAS offers a multidimensional approach to bodily interaction in terms of physical activity, comprising affective, cognitive, and behavioral aspects. Specifically, the items that constitute the EPAS show a high internal consistency (Säfvenbom et al., 2016; Mikalsen et al., 2019), which gives a strong indication for physical activity as a type of behavior that includes mental, biological, and social systems. This scale has nine items, such as the person's desire to be physically active and the person's delight, meaning-, and identity-making in/through physical activity, as well as behavioral aspects, such as the person's willingness to sacrifice to maintain physical activity in the future. The items are designed as statements, such as “I am always going to be physically active” and “I think that physical activity is one of the most meaningful things to do.” The participants responded on a Likert-type scale ranging from 1 (disagree completely) to 7 (agree completely). Since the validation of EPAS in 2016 (Säfvenbom et al., 2014), EPAS has been applied to different samples of Norwegian adolescents in secondary and upper secondary schools (Kolle et al., 2017; Mikalsen et al., 2019). These studies confirm high internal consistency with a Cronbach's alpha above 0.9, thus indicating a reliable measurement model.

PAC was measured employing five items from Harter's Self-Perception Profile for Adolescents (Harter, 2012). These five items have been edited and translated into Norwegian by Wichstrøm (1995). The items were designed as statements, such as, “I'm better at sports than others my age,” and four alternative responses, where 1 is “agree very little” and 4 is “agree very much.” Harter's Self-Perception Profile instrument has been previously used in several studies of children's and adolescents' PAC (Balaguer et al., 2012; Säfvenbom and Jordalen, 2017; Eberline et al., 2018).

PPS was measured employing six items modified from a prior study measuring parental support for movement activities (Säfvenbom et al., 2013). These were designed as statements, such as, “Dad has always supported my physical activity,” and seven alternative responses, where 1 is “disagree completely” and 7 is “agree completely.”

Due to uncertainties regarding the impact of sex on 13- to 15-year-old adolescents' PA behavior, sex is applied as a sociodemographic control variable for MVPA in our analysis.

### Data Analysis

Table 1 presents an overview of the sample distribution of completed questionnaires and accelerometer measurements at the three consecutive data-collection time points.

**Table 1:** Completed questionnaires and accelerometer measurements at time points 1, 2, and 3.

**Year**	**Survey, *n* (boys/girls)**	**Accelerometer, *n* (boys/girls)**
2017	319 (160/159)	306 (150/156)
2018	259	228 (111/117)
2019	289	160 (64/96)

As shown in Table 1, the distribution of answers differs at all three measurement time points. Within the sample (*n* = 320 in 2017), 5.8% (*n* = 19) answered the questionnaire once, 27.1% (*n* = 89) answered the questionnaire twice, and 67.1% (*n* = 220) answered the questionnaire at all three time points. Further, 32.2% (*n* = 102) had valid accelerometer data at all three time points, and 37.5% (*n* = 119) and 32.2% (*n* = 96) had valid accelerometer data at, respectively, two and one measurement time points.

When performing Little's Missing Completely at Random Test to screen for missing data (SPSS, version 24; IBM Corporation, New York, USA), the results indicated that the data were not missing completely at random (χ^2^ = 1476.14, *df* = 1355, *p* = 0.011). Consequently, an independent sample *t*-test was conducted to explore possible differences between participants who had responded at all three measurement time points and those who had responded at only one or two time points. However, only one of 12 latent variables was found to be significantly different between the “complete responders” and the “not complete responders”: PAC at T2 (*p* = 0.49). The score of the complete responders was higher than the not complete responders (*M* = 2.55, *SD* = 0.69 vs. *M* = 2.30 *SD* = 0.70).

Frequency analysis indicates that all items were normally distributed at all three measurement time points with skewness ranging from −1.84 and 0.385 and kurtosis ranging from −0.88 and 3.11. (Lumley et al., 2002; Kline, 2011; Byrne, 2012; Normal distribution within the range of 3.00–10.00).

A measurement invariance test of the latent variables was conducted in M*plus* (version 8, Muthén and Muthén, 1998-2017; Table 2) to analyze the measurement instrument's ability to measure the same concept across different time points (Byrne, 2012). First, the scale factor structure was assessed by confirmatory analysis (CFA) at T1 with maximum likelihood estimation (MLR) using the following model fit indices: The comparative fit index (CFI) ≥ 0.90, the standardized root mean square residual (SRMR) ≤ 0.08, and the root mean square error approximation (RMSEA) ≤ 0.06 (Brown and Kenny, 2006). All variables demonstrate an acceptable fit except for PPS. In order to improve the fit indices for this variable, it was decided to split PPS into two new variables: perceived mother support (PMS) and perceived father support (PFS). This adjustment led to model indices that were acceptable. Further, measurement over time was assessed using the following criteria to test, respectively: weak factorial invariance (ΔCFI < 0.01, ΔRMSEA < 0.015, and ΔSRMR < 0.03) and strong factorial invariance (ΔCFI < 0.01, ΔRMSEA < 0.015, and ΔSRMR < 0.01). For the variables EPA and PAC, the test indicated acceptable fit indices for all three time points. For PMS and PFS, there was a need for letting one intercept free to obtain good fit indices and thereby establishing partial strong invariance for these constructs (Little, 2013).

**Table 2:** Model fit statistics for the test of measurement invariance at T1, T2, and T3.

**Variable**	**Model tested**	***X*^2^**	**df**	***p*-value**	**RMSEA**	**ΔRMSEA**	**CFI**	**ΔCFI**	**SRMR**	**ΔSRMR**
Eagerness	Configural invariance	536.46	225	0.000	0.065 (0.058–0.072)	–	0.950	–	0.037	–
	Weak invariance	571.15	239	0.000	0.065 (0.058–0.072)	–	0.947	−0.003	0.052	0.022
	Strong invariance	617.70	253	0.000	0.066 (0.060–0.073)	0.001	0.942	−0.005	0.055	0.003
Perceived athletic competence	Configural invariance	34.69	15	0.003	0.063 (0.036–0.091)	–	0.984	–	0.031	–
	Weak invariance	40.38	19	0.003	0.059 (0.033–0.084)	−0.004	0.983	−0.001	0.044	0.013
	Strong invariance	47.38	23	0.288	0.057 (0.033–0.080)	−0.002	0.980	−0.003	0.046	0.002
Perceived mother support	Configural invariance	27.60	15	0.024	0.051 (0.018–0.080)	–	0.989	–	0.039	–
	Weak invariance	31.47	19	0.036	0.045 (0.012–−0.071)	−0.006	0.989	–	0.040	0.001
	Strong invariance	54.35	23	0.132	0.057 (0.033–0.081)	−0.012	0.981	−0.008	0.044	0.004
Perceived father support	Configural invariance	8.93	15	0.881	0.000 (0.000–0.026)	–	1.000	–	0.025	–
	Weak invariance	12.49	19	0.863	0.000 (0.000–0.026)	–	1.000	–	0.031	0.006
	Strong invariance	27.28	23	0.922	0.000 (0.000–0.040)	0.014	1.000	–	0.035	0.004

*The test of weak factorial invariance used the following criteria: X^2^ > df, RMSEA < 0.08, ΔRMSEA < 0.015, CFI > 0.90, ΔCFI < 0.01, SRMR < 0.08, ΔSRMR < 0.03 (ΔSRMR < 0.015; strong invariance). The test of strong invariance used ΔSRMR < 0.015 (Sass, 2011)*.

LCGA is a person-centered method, suited for the estimation of between-person differences in within-person change, often referred to as trajectories (Isiordia and Ferrer, 2018). Latent growth modeling, such as LCGA, is, according to Curran et al. (2010), characterized by high levels of statistical power and as highly flexible because of the ability to incorporate complexities, such as partially missing data, non-linear change, unequal time points, and heterogeneous growth processes. LCGA was, therefore, conducted (using M*plus*) on the collected PA data from all three time points to explore for latent classes of adolescents with similar trajectories of PA from 13 to 15 years old. The estimates of variance and covariance for the growth factor, PA, were fixed to zero, assuming that all growth trajectories within each class were identical. To explore the number of latent trajectories within the total sample, a stepwise model comparison approach was conducted to compare a one-class model to models with successively more classes (Jung and Wickrama, 2008).

A combination of criteria was applied to guide the decision on the number classes within the sample, comprising finding the model with the smallest Aikaike information criterion and Bayesian information criteria. Further, the Lo–Mendell–Rubin likelihood ratio test (L-M-R) and the bootstrap likelihood ratio test (BLRT) (Nylund et al., 2007; Jung and Wickrama, 2008) were used to compare improvement in fit between neighboring classes. Significant *p*-values in both L-M-R and BLRT indicate that a k-1 class model is rejected in favor of the k class model (Nylund et al., 2007). Additionally, theoretical justification, interpretability, class-size (>5%) (Jung and Wickrama, 2008) and class solutions with >75 participants in a profile were considered (Lubke and Neale, 2006). The value of entropy (without being assessed as a measure of fit; Ramaswamy et al., 1993) was taken into account when evaluating the estimation probability of likely class membership for participants. The entropy ranges from 0 to 1, where higher entropy indicates higher class separation. There is no clear consensus about the acceptable cutoff for what is recommended entropy, yet extremely low entropy might indicate models that are not useful for their purpose (Feldman et al., 2009).

We proceeded to conduct a series of analyses to explore whether there were differences between the trajectories related to distal outcome variables at all three time points (EPA, PAC, PFS, PMS). We applied Bolck et al. (2004) BCH three-step method, which is assessed to be the most robust and flexible omnibus test that includes differences between the two classes on each distal outcome variable (Bakk and Vermunt, 2016). Effect sizes were calculated for the differences in the distal outcomes between the trajectories, using Cohen's *d* (Cohen, 1988).

## Results

The results are presented according to the two main aims of this study: an exploration of valid trajectories in PA behavior in adolescents and an examination of the probable ways in which the distal outcome variables relate to the different PA trajectories within the sample.

### PA Behavior and Latent Trajectories in the Sample

The mean PA level in the total sample at T1, T2, and T3 were, respectively, 90.45 (*n* = 300), 72.00 (*n* = 175), and 66.00 (*n* = 163) min/day. Participants who fulfill the national PA recommendations were in T1, T2, and T3, respectively, 87, 63, and 47%. By applying a stepwise comparison of the LCGA, a solution with two different classes within the total sample was favored.

Deciding for the two-class model against the three-class model was based on both fit statistics and substantive considerations (Feldman et al., 2009; Table 3). The two-class model gained the lowest BIC value and significant results for the BLRT and L-M-R. Further, the two classes consisting of, respectively, 26 and 74% of the total sample were considered to be of sufficient size and theoretically sound, and the two identified trajectories within the graphical data plot were judged to be logic PA trajectories considering existing knowledge about PA development among adolescents.

**Table 3:** Fit indices for latent class growth models of MVPA.

**No. of trajectories**	**No. of free par**.	**AIC**	**BIC**	**BLRT (*p*)**	**L-M-R (*p*)**	**Entropy**	**Latent class size: *n* (%)**
1	5	6911.11	6929.97				321 (100)
2	8	6971.35	6901.52	0.000	0.000	0.53	83/238 (26/74)
3	11	6869.68	6911.16	0.429	0.05	0.57	215/14/92 (67/04/29)
4	14	6870.08	6922.88	0.364	0.373	0.69	5/76/226/14 (2/24/70/4)

*N = 321. AIC, Akaike information criterion; BIC, Bayesian information criterion; BLRT, bootstrap likelihood ratio test; L-M-R, Lo-Mendell-Rubin adjusted likelihood ratio test*.

The entropy of the two-class solution was 0.53, which is in the lower range of what could be considered acceptable and, therefore, indicates some uncertainty concerning class membership. This is further discussed in the limitations.

**Figure 1:**
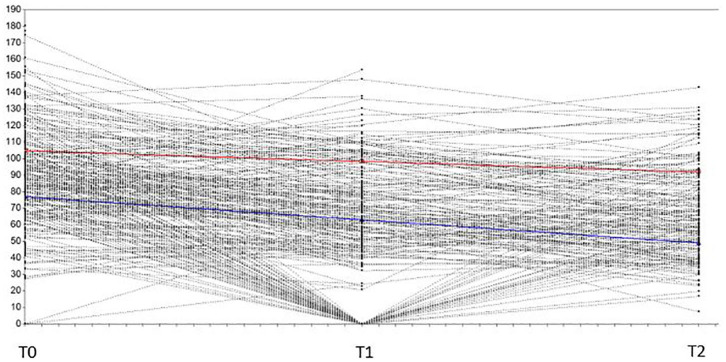
The two trajectories related to PA at baseline (seventh grade), follow-up (eighth grade), and posttest (ninth grade). *X*-axis = Data collection time points (T0 = seventh grade, T1 = eighth grade, T2 = ninth grade); *y*-axis = PA level (MVPA/day).

The identified classes represent two different and meaningful course trajectories of physical activity behavior in adolescents from seventh to ninth grade (Figure 1).

Trajectory 1 (prevalence: *n* = 83, 26% of the total sample) is labeled “decrease from very high” and includes subjects with a very high baseline level of PA and with scores slightly decreasing over a period of 2 years (intercept: *M* = 104.79, *SE* = 3.53, *p* < 0.001; slope: *M* = −6.4, *SE* = 2.92, *p* = 0.029).

Trajectory 2 (prevalence: *n* = 238, 74% of the total sample) is labeled “steeper decrease from high” and includes subjects with high baseline levels of PA and with scores steeply decreasing over a period of 2 years (intercept: *M* = 77.06, *SE* = 2.51, *p* < 0.001; slope: *M* = −13.98, *SE* = 1.31, *p* < 0.001).

### Distal Outcome Variables Related to the Underlying Pattern of PA

The trajectories demonstrate a linear pattern across the three time points. Hence, the difference between the two trajectories on the distal outcome variables was analyzed at baseline (T1), follow-up (T2), and posttest (T3). Several sets of analyses, which apply to the BCH-method, were carried out to assess whether the distal outcome variables (EPA, PAC, PFS, PMS) differ across the two trajectories (Table 4).

**Table 4:** Distal outcome variables at T1, T2, and T3.

**Distal outcome variables**	**Trajectory 1** ***n* = 83, 26%** ***M/SD***	**Trajectory 2** ***n* = 283, 74%** ***M/SD***	**1 vs. 2** ***X*^2^/*p*-value**	**1 vs. 2** **Cohen's d ES**
T1 Eagerness	6.32 (1.25)	5.46 (1.70)	19.31/^***^	0.58
T1 Perceived athletic competence	2.88 (.85)	2.34 (.87)	19.77/^***^	0.63
T1 Perceived support mother	5.79 (1.54)	5.58 (1.80)	0.79/	0.13
T1 Perceived support father	5.92 (1.66)	5.57 (1.85)	2.09/	0.20
T2 Eagerness	6.22 (1.97)	4.93 (2.27)	20.02/^***^	0.61
T2 Perceived athletic competence	2.97 (1.00)	2.32 (1.01)	22.23/^***^	0.65
T2 Perceived support mother	5.67 (1.99)	5.41 (2.34)	0.74/	0.12
T2 Perceived support father	5.72 (2.20)	5.40 (2.25)	1.067/	0.14
T3 Eagerness	6.42 (1.28)	5.03 (2.02)	43.41/^***^	0.82
T3 Perceived athletic competence	2.89 (.80)	2.31 (1.13)	21.48/^***^	0.59
T3 Perceived support mother	5.63 (1.90)	5.36 (2.14)	0.90/	0.13
T3 Perceived support father	5.78 (1.97)	5.31 (2.22)	2.52/	0.22

*p-value*:

* *< 0.05*,

** *< 0.01*,

*** *< 0.001*.

*Cohen's d effect size (ES): 0.01–0.19 (very small), 0.20–0.49 (small), 0.50–0.79 (moderate), 0.80–1.19 (large), 1.20–1.99 (very large), and 2.00 (huge). T1, baseline measurement (2017); T2, follow up measurement (2018); T3, post measurement (2019)*.

The scores of trajectory 1, “decrease from very high,” on EPA were overall high throughout the three measurement time points (T1: *M* = 6.32, *SD* = 1.25; T2: *M* = 6.22, *SD* = 1.97; and T3: *M* = 6.42, *SD* = 1.28; Table 2). The scores of trajectory 2, “steeper decrease from high,” on EPA were also high. However, they report values, on average, 1.2 points below the adolescents in trajectory 1 and reveal a more noticeable decline in EPA from T1 to T3 (*M* = 5.46, *SD* = 1.7; *M* = 4.93, *SD* = 2.27; and *M* = 5.03, *SD* = 2.02).

The scores on PAC were relatively consistent in both trajectory groups throughout the three measurement time points (Trajectory 1 at T1: *M* = 2.88, *SD* = 0.85; at T2: *M* = 2.97, *SD* = 1.00; at T3: *M* = 2.89, *SD* = 0.80; trajectory 2 at T1: *M* = 2.34, *SD* = 0.87; at T2: *M* = 2.32, *SD* = 1.01; at T3: *M* = 2.31, *SD* = 1.13).

The scores of both EPA and PAC were significantly different in the two trajectories at all three measurement time points (Table 2). Cohen's *d* effect sizes (ES) were moderate in both EPA and PAC at all measurement time points (0.58–0.65) except for a large ES on the differences in EPA between the two trajectories in T3 (0.82).

The mean scores in both trajectories show weak declines on perceived support from both father and mother from T1 to T3 (Table 4), but the differences in both PMS and PFS between trajectory 1 “decrease from very high” and trajectory 2 “steeper decrease from high” were not significant at any measurement time point and the effect size was in the range of very small to small.

Analysis of whether it was likely that the proportion of sex differed between the two trajectories showed no significant differences.

## Discussion

### The Two Different PA Trajectories

The person-centered analysis revealed two different PA trajectories within the total sample. The two trajectories, labeled trajectory 1 (TRA1) “decrease from very high” and trajectory 2 (TRA2) “steeper decrease from high” differed in several ways. First, TRA1 consisted of 26% of the sample, and the medium active group consisted of the remaining 74%. Both groups had a high PA level at T1 (Steene-Johannesen et al., 2019); however, TRA1 had a higher PA level at both baseline and throughout the three measurement time points than TRA 2, respectively, 105 vs. 77 min MVPA daily at baseline. Thus, most of the adolescents in both trajectories fulfilled the national health recommendation at baseline, which is not in line with previous Norwegian mapping studies of adolescents' PA level (Steene-Johannesen et al., 2019). The Norwegian study did, however, measure the PA level in 9- and 15-year-olds, and T1 in this study measured 12- to 13-year-olds. Adolescents entering their teens are in a transitional phase, meaning that they may still carry with them the playful mindset of the childhood culture (Mikalsen and Lagestad, 2018) and, at the same time, experience increased abilities and possibilities of moving around in their local communities (Helse- og omsorgsdepartementet, 2004). A study by Lam and McHale (2015) reveals an increase in leisure-time PA during middle childhood (with a peak for girls at 12 and for boys at 13 years), thus supporting a possible tendency of children at the beginning of adolescence to be more physically active than those of both lower and higher age.

Both trajectories reveal a significant decline in PA level from T1 to T3 (average decline accordingly in TRA1: 6.4 and in TRA2: 13.98 min of MVPA/day/year). The decline in daily MVPA, shown in this study, is above the Norwegian mapping study by Steene-Johannesen et al. (2019), who found a decline of 3.5 min/day from 6- to 15-year-olds (2.5/3 min from 9- to 15-year-olds). However, another follow-up study showed a linear MVPA decline of 8.5 min/day from 14- to 19-year-olds among Norwegians (Lagestad et al., 2018). Differences in PA trajectories may be indicators of the wide range of variables, which have different kinds of impact on different people at different ages and contexts (Cooper et al., 2015). Throughout the three measurement time points, TRA2 showed a greater decline in the PA-level than TRA1 (respectively, 77, 63, and 49 vs. 10, 98.5, and 92 min daily MVPA). This gives support to previous research finding previous PA to be a predictor of future PA and to the tendency of a PA level to decline in the majority of the population throughout their teens (Craggs et al., 2011; Rangul et al., 2011; Uijtdewilligen et al., 2011). By using person-centered analysis, as in the current study, opportunities to provide a more nuanced picture of the PA behavior in adolescents are present. Person-centered analysis with a larger sample than this study sample would probably be able to identify further nuances of PA behavior within the population of adolescents.

### The Distal Outcome Variables Association to the Different PA Trajectories

The adolescents in TRA1 reported higher mean levels of EPA and PAC as seventh, eighth, and ninth graders. This is in line with previous research, indicating the possibility of EPA to predict sustainable PA involvement and the promotive impact of PAC on adolescents' PA behavior (Säfvenbom et al., 2016; Mikalsen et al., 2019).

Regarding EPA, both TRA1 and TRA2 reveal high values at all measurement time points. However, TRA1 is associated with average EPA values 1.2 points higher than TRA2. Furthermore, the effect size of differences between TRA1 “decrease from very high” and TRA2 increased from baseline (seventh grade) to posttest (ninth grade). This finding indicates a stronger experience of PA as enjoyable, personally relevant, and self-confirming behavior (i.e., “I regard myself as a person who exercises”) within the most active adolescents and even stronger as they get older. The potential relationship between affective reasons to be involved in PA and a continuing high PA level may, therefore, be assumed strengthened by the findings of the current study. Accordingly, several recent theoretical and empirical contributions are pointing in the direction of affect-related experiences, closely associated with the behavior, as significantly important in deciding whether to be involved in PA or not (Nasuti and Rhodes, 2013; Dishman et al., 2018). In some studies, affect-related experiences are even considered as superior to rational cognitive-related reasons to become genuinely involved in PA (Paxton et al., 2004; Blankenship and Ayers, 2010; McBain, 2013; Beni et al., 2017). Ekkekakis and Dafermos (2012) even propose an upcoming paradigm shift from a rational cognitive paradigm to a hedonistic paradigm to understand why some are physically active and some less so. They claim with the words of Richard A. Friedman and Fred Charatan that “The real value of it is not in the terms of abstract health benefits like longevity—but because it feels good when you do it or when it's over. To hell with Hygeia, the truth lies in the pleasure” (Ekkekakis and Dafermos, 2012, p. 295).

Acknowledging that EPA is also a concept based on both hedonistic and eudaimonic emotions concerning PA behavior, our results support research that highlights the impact of personally significant meaning in the process of implementing PA as a habitual behavior (Kretchmar, 2007; Beni et al., 2017; Dishman et al., 2018). According to Metheny (1968, p. 5), things “are made personally meaningful as we seize upon it, take it into ourselves and become involved with it. The extent of involvement in PA can, thus, be interpreted as a symptom of finding PA meaningful or significant.”

The scores on PAC were relatively consistent in both TRA1 and TRA2 through the three measurement time points. However, the higher level of PAC in TRA1 indicates that perceiving higher athletic competence is related to a higher level of MVPA behavior and, further, that a stable score on PAC through all three measurement time points may contribute to lessening the decline of MVPA during the same period. The lower baseline levels of PAC in TRA2, which, however, also remained stable through T1–T3, may be interpreted as a significant correlate to both a lower MVPA level and a more apparent decline in the participants' MVPA through T1–T3. These results substantiate that perception of competence in physical activity contexts is related to physical activity behavior as found in several other studies (De Meester et al., 2016; Timo et al., 2016; Eberline et al., 2018).

A possible way of interpreting the relation of PAC and the adolescents' PA level in the two different trajectories is to assess PAC as a construction made by an individual–context interaction. This construction constrains a unified understanding of what to be competent in and which skill level is within the socially acknowledged range (Larsson, 2016). Perception of competence may, thus, become a question of negotiation with the culturally constructed constraints of the activity (Mikalsen and Lagestad, 2019). In Norway, 89% of all young people report former involvement in organized sports during childhood and adolescence (Bakken, 2019). Participating in organized sports will, in this perspective, stand out as a context for negotiation of perceived athletic competence—a context that is familiar for many adolescents in contemporary societies. However, a new population study among Norwegians shows that the apex of dropout from organized sports for girls and boys is 13 and 14 years, respectively (HuNT, 2019). Reaching high school, only 40% of adolescents continue to participate in organized sports (Bakken, 2019). This may imply a probability of adolescents dropping out of sports due to experiences of not being able to perform well enough in their sport (Säfvenbom et al., 2016; Seippel et al., 2016; Bakken, 2019). Interpretation of the steep MVPA decline in TRA2 might be in line with these findings.

Our findings did not reveal significant differences in PMS and PFS between TRA1 in TRA2 at any time point. According to Bailey (2018), any interrelated system concerning PA has the potential to foster development and learning in the individual. Perception of support from proximal social relations, such as parents, is an interrelated system that, in several research contributions, is revealed to be of significance for their offspring's PA behavior (Fredricks and Eccles, 2004; Weiss and Phillips, 2015). The findings in the present study support these findings. Our findings indicate that parents are perceived as supportive regardless of the extent of the adolescents' PA behavior. However, our results may, in some way, imply a propensity, which also is suggested by Beets et al. (2010, p. 635), for parental support of PA to fluctuate with their offspring's involvement in PA contexts; more active adolescents perceive more family support. Nevertheless, although perceived support from parents has different kinds of impact on the adolescents' PA behavior (Weiss and Phillips, 2015; Osher et al., 2018), previous research finds the amount of explained variance for PA to be generally small as can be said to be supported by this study.

### Strengths and Limitations

This study of adolescents' PA level, is a longitudinal/prospective cohort study as recommended by Atkin et al. (2016) to optimize our understanding of the coactions of interrelated variables. According to the recommendations of Atkin et al. (2016), we have employed objective PA measurements (Actigraph GT1M) to identify the participants' PA level, which is assessed to be a more accurate method than self-reporting PA (Lund-Blix et al., 2017). The theoretical framework in this study applies to the RDS meta-theory (Lerner et al., 2018), which is recommended (Atkin et al., 2016) for capturing the context, timing, and complexity of adolescents' PA.

The study has some limitations. The LCGA identified two meaningful PA trajectories, but the entropy value was relatively low, consequently indicating uncertainty relating to participants' class membership (Feldman et al., 2009). The dropout rate was handled with FIML (Muthén et al., 1987; Cham et al., 2017), and the group size is, according to Jung and Wickrama (2008) and Lubke and Neale (2006), considered sufficient. We argue, though, that future studies should involve larger samples to replicate the findings of the current study as larger sample sizes are likely to result in stronger entropy (Feldman et al., 2009). However, the BCH analysis, which reveals significant differences in the expected direction in the distal outcome variables EPA and PAC, with moderate-to-large effect sizes, can strengthen our argument for the two trajectories being different from one another. Furthermore, we are aware of limitations in the study concerning the accelerometer's ability to measure horizontal activities, such as biking and swimming. However, at T1, data from the questionnaire revealed that 83.3% of the participants had not been to the swimming pool during the week they wore the accelerometer, 8.8% had been to the swimming pool once, and 7.9% had been to the swimming pool twice or more. There are reasons to believe that both swimming and biking probably would have increased the overall PA measurement but nevertheless not by enough to alter the interpretations of the participants' PA level and the covariance between the PA level and the distal outcome variables. Finally, the PA measurement is related to random weekdays. Although the Norwegian mapping study of PA behavior reveals a lower PA level in adolescents during weekends (Steene-Johannesen et al., 2019), a possible bias in the present study's PA measurement can be moderated by the random day PA registration.

### Concluding Remarks

The person-centered analysis reveals two different PA trajectories within the total sample. Trajectory 2, “steeper decrease from high,” consisting of the majority (74%) of the sample, had a lower PA level at baseline and a following steeper PA decline through follow-up PA measurements in eighth and ninth grade, than the smaller Trajectory 1, “decrease from very high,” (26%) with a higher PA level at baseline and a less steep decline from seventh to ninth grade.

The values of EPA and PAC were confirmed to be significantly higher in trajectory 1, “decrease from very high,” than in trajectory 2, “steeper decrease from high.” In other words, adolescents who were more physically active in the seventh, eighth, and ninth grade were more eager to be involved in PA, and they perceived themselves as more athletically competent than the less physically active adolescents in trajectory 2, “steeper decrease from high.”

Our results confirm the findings of several prospective studies, showing that people's PA levels fluctuate through different periods of life and that transitional phases, such as the transition from childhood to adolescence, might be particularly instable. The practical implications of the study are that parents, teachers, trainers, and other professionals should strive to optimize environmental factors that increase adolescents' experience of eagerness for physical activity and adolescents' experiences regarding feeling physical athletically competent. Future research should continue to investigate the impact of eagerness for physical activity on adolescents' involvement in different physical activity contexts.

## Data Availability Statement

The datasets generated for this study are available on request to the corresponding author.

## Ethics Statement

The study has been approved by the Ethics Committee of the ‘Norwegian Centre for Research Data (NSD)’, and both the parents and adolescents have given their written informed consent to participate.

## Author Contributions

HM has gathered the empirical material for this article. HM has in cooperation with MB and RS worked out the article design. HM has done the review of previous research and further done the writing of all parts of the article. MB has done the analysis of the empirical material and constructed the tables and the figure. PL and RS has read the article and cooperated in writing all sections of the article. All authors contributed to the article and approved the submitted version.

## Conflict of Interest

The authors declare that the research was conducted in the absence of any commercial or financial relationships that could be construed as a potential conflict of interest.
